# Herbicide toxicity to *Nitrospirillum amazonense* : assessing bacterial survival and microbial functionality

**DOI:** 10.1002/ps.70330

**Published:** 2025-10-28

**Authors:** Luana Carolina Gomes Jonck, Patrícia Andrea Monquero, Márcia Maria Rosa Magri, Carina de Fátima Felippe

**Affiliations:** ^1^ Center of Agricultural Sciences – Federal University of São Carlos (UFSCar) Araras Brazil

**Keywords:** biological nitrogen fixation, phytohormones, plant growth‐promoting bacteria, toxicity

## Abstract

**BACKGROUND:**

Herbicides can exert significant toxicity on nontarget organisms, including plant growth‐promoting bacteria. *Nitrospirillum amazonense* has been shown to be highly effective in enhancing sugarcane growth through biological nitrogen fixation (BNF) and the production of the phytohormone indole‐3‐acetic acid (IAA). However, the combined effects of herbicide application and this microbial technology remain poorly understood, representing a critical knowledge gap for sustainable sugarcane management. Therefore, the objective of this study was to evaluate, through four *in vitro* assays, the impact of nine pre‐emergent herbicides on the survival and physiological functions of *N. amazonense*.

**RESULTS:**

Herbicides exerted differential and molecule‐specific effects on *N. amazonense*. Indaziflam, metribuzin, *S*‐metolachlor and sulfentrazone caused no detrimental impacts on bacterial performance and, in some cases, even enhanced cell growth, IAA production and/or BNF. Isoxaflutole promoted growth at sublethal concentrations but became toxic at higher doses, while leaving IAA production and BNF unaffected. Clomazone and imazapic reduced bacterial growth yet did not compromise soil survival and stimulated both IAA and BNF. Flumioxazin and tebuthiuron enhanced growth and BNF but reduced IAA production.

**CONCLUSION:**

This study provides novel evidence that herbicides induce complex and specific modulations in *N. amazonense*, influencing not only bacterial survival but also the physiological mechanisms underlying plant growth promotion. Such specificity underscores the importance of herbicide selection in integrated management systems. The identification of compatible herbicides enables strategic combinations that sustain weed control while preserving microbial benefits, thereby advancing sustainable sugarcane production. © 2025 The Author(s). *Pest Management Science* published by John Wiley & Sons Ltd on behalf of Society of Chemical Industry.

## Graphical abstract

Herbicides elicit a spectrum of responses in *Nitrospirillum amazonense*, ranging from the stimulation of metabolic functions (biological nitrogen fixation, IAA production) to significant growth inhibition and acute toxicity.

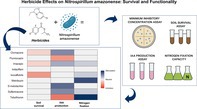

## 1 INTRODUCTION

Herbicides are indispensable tools in modern conventional agriculture and are widely adopted in production systems worldwide because of their efficiency in weed control, high operational yield and lower cost per area than alternative methods.^1^ In sugarcane cultivation, the use of pre‐emergent herbicides—applied directly to the soil before crop emergence—is a well‐established practice for weed management.^2^

In parallel, biotechnological advances have introduced sustainable alternatives to the sugarcane sector, with plant growth‐promoting bacteria (PGPB)‐based bioinoculants emerging as promising tools. This approach aims to increase productivity while reducing synthetic input dependency, aligning with growing sustainability demands in the sugar‐energy industry.^3^

The agronomic benefits of PGPB are mainly mediated through two key mechanisms. First, biological nitrogen fixation (BNF) allows diazotrophic bacteria to reduce atmospheric N₂ into plant‐assimilable ammonia via the nitrogenase enzyme complex, a process that can partially or even fully replace synthetic nitrogen fertilizers.^4^ In sugarcane, BNF is estimated to supply ≤70% of the crop's nitrogen demand.^5^ Second, PGPB produce phytohormones, particularly auxins such as indole‐3‐acetic acid (IAA), which can reprogram plant development.^6^ Inoculation studies have shown increases of ≤51% in root length and 70% in root volume in sugarcane seedlings, significantly enhancing establishment and nutrient uptake.^7^ Among these microorganisms, *Nitrospirillum amazonense* has emerged as a valuable biotechnological ally, with demonstrated capacities to accelerate bud sprouting, modify root architecture, and increase stalk yield by up to 18%.^8^

However, the interaction between these two strategies—chemical weed control and microbial plant growth promotion—remains poorly understood. Although herbicides are designed to target specific biological mechanisms, numerous studies have demonstrated unintended effects on nontarget organisms.^9^ Soil microbial communities are especially vulnerable and are affected by direct toxicity or disruptions in key biochemical processes.^10^ The mechanisms involved include protein synthesis inhibition, DNA damage and membrane lipid peroxidation.^11^

Studies focusing on PGPB have revealed bactericidal and bacteriostatic effects on species such as *Gluconacetobacter diazotrophicus* and *Azospirillum Brasiliense* upon herbicide exposure.^12^, ^13^ These effects compromise not only microbial survival, but also critical ecological functions, including BNF and phytohormone synthesis.^14^ This issue is relevant in the context of the increasing use of microbial inoculants as sustainable alternatives to chemical fertilizers.

Given this scenario, the present study aimed to assess the ecotoxicological effects of nine pre‐emergent herbicides commonly used in sugarcane fields on the survival and functional activity of *N. amazonense*. Using four *in vitro* assays—minimum inhibitory concentration testing, soil survival analysis, BNF quantification and IAA production—we tested the hypothesis that these chemical compounds can impair both the viability and functionality of this important rhizobacterium. Our findings can inform integrated management strategies that reconcile chemical weed control with microbial plant growth promotion.

## 2 MATERIALS AND METHODS

### 2.1 Bacterial inoculum preparation

The *N. amazonense* strain (BR 11145 ‐ CBAmC) was obtained from the diazotrophic bacterial collection at Embrapa Agrobiology. Bacterial cells were activated and cultured in 200 mL nutrient broth (NB) composed of the following (in g L^−1^ distilled water): 1.0 meat extract, 2.0 yeast extract, 5.0 peptone and 5.0 sodium chloride. The culture was incubated in a shaker at 30 °C and 150 rpm for 24 h until an optical density at 600 nm (OD₆₀₀) >0.8 was reached, which is equivalent to approximately 2 × 10^9^ colony‐forming unites (CFU) mL^−1^.

### 2.2 Herbicide solution preparation

Nine pre‐emergent herbicides registered for use in sugarcane crops were selected (Table 1). Stock solutions were prepared from the commercial formulations of each herbicide and diluted such that the addition of 1 mL of the solution to the culture medium resulted in the desired final concentrations.

**Table 1:** List of evaluated herbicide treatments

Treatment		Dose	
Active ingredient	Commercial name	g L^−1^ a.i. ha^−1^	g L^−1^ c.p. ha^−1^
Control	‐	‐	‐
Clomazone	Gamit	720	1440
Imazapic	Plateau	200	285
Tebuthiuron	Combine 500 SC	800	1600
Indaziflam	Alion	75	150
*S*‐metolachlor	Dual gold	1680	1750
Metribuzin	Sencor 480	1680	3500
Isoxaflutole	Provence 750 WG	150	200
Sulfentrazone	Boral 500 SC	800	1600
Flumioxazin	Flumyzin 500 SC	150	300

† a.i. = active ingredient, c.p. = commercial product.

### 2.3 Evaluation of the minimum inhibitory concentration (MIC)

The experiment was conducted in a completely randomized design with a 9 × 5 factorial arrangement with three replicates. The factors consisted of nine pre‐emergence herbicides (Table 1) and five concentrations: the recommended commercial dose (CD), 2× CD, 1.5× CD, 0.5× CD and 0.25× CD, alongside an untreated control.

One milliliter (1 mL) of each herbicide stock solution was added to 125‐mL Erlenmeyer flasks containing 50 mL NB. For the control treatment, an equivalent volume of sterile distilled water was used. Each flask was subsequently inoculated with 0.1 mL of the bacterial inoculum. The flasks were then incubated on a rotary shaker at 150 rpm and 30 ± 2 °C for 48 h.

After incubation, the population density of *N. amazonense* was quantified by determining the CFUs. This was performed by serially diluting the cultures up to 10^−8^ in a 0.85% saline solution, followed by plating via the spread‐plate technique. For each dilution, 100‐μL aliquots were plated in triplicate onto Petri dishes with nutrient agar and incubated at 30 °C for 24 h.

The CFU count per milliliter was calculated via the following formula:

$$\mathrm{CFU}\;{\mathrm{mL}}^{-1}=\left(\text{number of colonies}\times \text{dilution factor}\right)/\text{volume plated}\;\left(\mathrm{mL}\right)$$

### 2.4 Survival of *N. amazonense* in herbicide‐treated soil

The experiment was conducted in a completely randomized design with four replicates. The survival of *N. amazonense* was evaluated in soil treated with nine herbicides at their respective commercial doses (Table 1). The design also included two controls: an unamended control (no herbicide, no inoculum) and an inoculated control (inoculum only).

Soil samples (1000 g) from a native forest area were crushed, sieved (2‐mm mesh), homogenized, and sterilized by tyndallization. This process involved autoclaving the soil for 20 min on three consecutive days.

Herbicide solutions were sprayed onto sterilized soil at a spray volume equivalent to 200 L ha^−1^. The microbial inoculant was then applied at a volume equivalent to 150 L ha^−1^. For the control treatments, sterile distilled water was applied at the same volume. The treated soil samples were incubated at room temperature for 48 h.

After incubation, a 10 g soil sample from each treatment was suspended in 90 mL saline solution. This suspension was serially diluted to 10^−6^ by transferring 1‐mL aliquots into tubes containing 9 mL saline solution. From each dilution, a 0.1‐mL aliquot was inoculated in triplicate into vials containing 5 mL semisolid, semiselective LGI medium. The vials were incubated at 30 °C for 7 days.

The bacterial population was quantified via the most probable number (MPN) method with McCrady's table for three replicates per dilution. Growth was confirmed by the formation of a characteristic pellicle on the surface of the medium.

### 2.5 Evaluation of IAA production

This experiment was arranged in a completely randomized design with three replicates. Nine herbicides (Table 1) were tested at their recommended field doses.

For each treatment, 125‐mL Erlenmeyer flasks containing 50 mL NB supplemented with 1 mg mL^−1^ tryptophan were inoculated with 0.1 mL *N. amazonense* inoculum. One milliliter (1 mL) of each herbicide stock solution was then added to the corresponding flask. The cultures were incubated on a rotary shaker at 150 rpm and 30 ± 2 °C for 48 h.

Following incubation, a 2‐mL aliquot of the culture was transferred to a microcentrifuge tube and centrifuged at 10 000 rpm for 10 min. One milliliter (1 mL) of the supernatant was mixed with 1 mL Salkowski's reagent (0.675 g FeCl₃ in 125 mL 35% perchloric acid). The mixture was incubated in the dark for 30 min.

The IAA concentration was determined spectrophotometrically by measuring the absorbance at 540 nm. The final values were calculated against a standard curve prepared with IAA concentrations ranging from 1 to 100 mg L^−1^.

### 2.6 Assessment of BNF capacity

This experiment was conducted in a completely randomized design with nine herbicide treatments (Table 1) and three replicates.

For each treatment, test tubes containing 10 mL nitrogen‐free bromothymol blue (NFB) medium were inoculated with 0.1 mL bacterial inoculum. One milliliter (1 mL) of each herbicide stock solution was added to the respective tubes. All the tubes were incubated in a BOD chamber at 30 ± 2 °C for 7 days.

The total nitrogen content was determined via the semimicro Kjeldahl method. Each tube received 0.7 g of a digestion mixture (100 g Na₂SO₄, 10 g CuSO₄·5H₂O and 1 g selenium powder), followed by the addition of 1 mL hydrogen peroxide (H₂O₂) and 2 mL concentrated sulfuric acid (H₂SO₄). The tubes were heated at 100 °C for 8 h, 180 °C for 2 h and finally at 360 °C until the digestate turned pale green.

After digestion, the samples were distilled with 40% sodium hydroxide (NaOH). The released ammonia was captured in an Erlenmeyer flask containing 25 mL boric acid (H₃BO₃) with a mixed pH indicator (methyl red and bromocresol green).

The resulting solution was titrated with 0.01 mol L^−1^ hydrochloric acid (HCl), and the total nitrogen (TN) content was calculated via the following formula:

$$\mathrm{TN}\;\left(\%\right)=\left[\left(V_{a}-V_{b}\right)\;\times \;F\;\times \;0.1\;\times \;0.014\;\times \;100\right]/P₁$$

where TN is the total nitrogen content in the sample (%); *V*ₐ is the volume of HCl used in sample titration (mL); *V*_b_ is the volume of HCl used in the blank titration (mL); *F* is a correction factor for 0.01 mol L^−1^ HCl; and *P*₁ is the sample mass (mL).

### 2.7 Statistical analysis

All data were initially tested for normality (Shapiro–Wilk test) and homogeneity of variances (Bartlett's test) to verify the assumptions for ANOVA. Upon confirmation of these assumptions, an ANOVA (*F*‐test) was performed at the 5% significance level (*P* < 0.05). Where significant differences were identified, treatment means were compared using Tukey's honestly significant difference (HSD) *post hoc* test.

For dose–response experiments, regression models were fitted to the data, and the results were visualized graphically. Model selection was guided by two primary criteria: the statistical significance (*P* < 0.05) of the model parameters (evaluated by Student's *t‐*tests for coefficients b₀, b₁, b₂ and b₃) and the coefficient of determination (*R* ^2^). Treatments that exhibited a response pattern characterized by stimulation at low doses followed by inhibition at higher concentrations were fitted to the Brain–Cousens hormesis model to estimate the parameters *b*, *d*, *e* and *f*.

All statistical analyses were conducted using R software (v4.5.1; R Core Team, 2025) within the rstudio integrated development environment (v2025.05.1 + 513; Posit PBC, Boston, MA, USA).

## 3 RESULTS

The growth of *N. amazonense* in response to different herbicides is presented in Figs 1, 2, 3, 4, 5, 6, 7, 8. The bacterial strain exhibited distinct responses depending on the specific herbicide.

**Figure 1:**
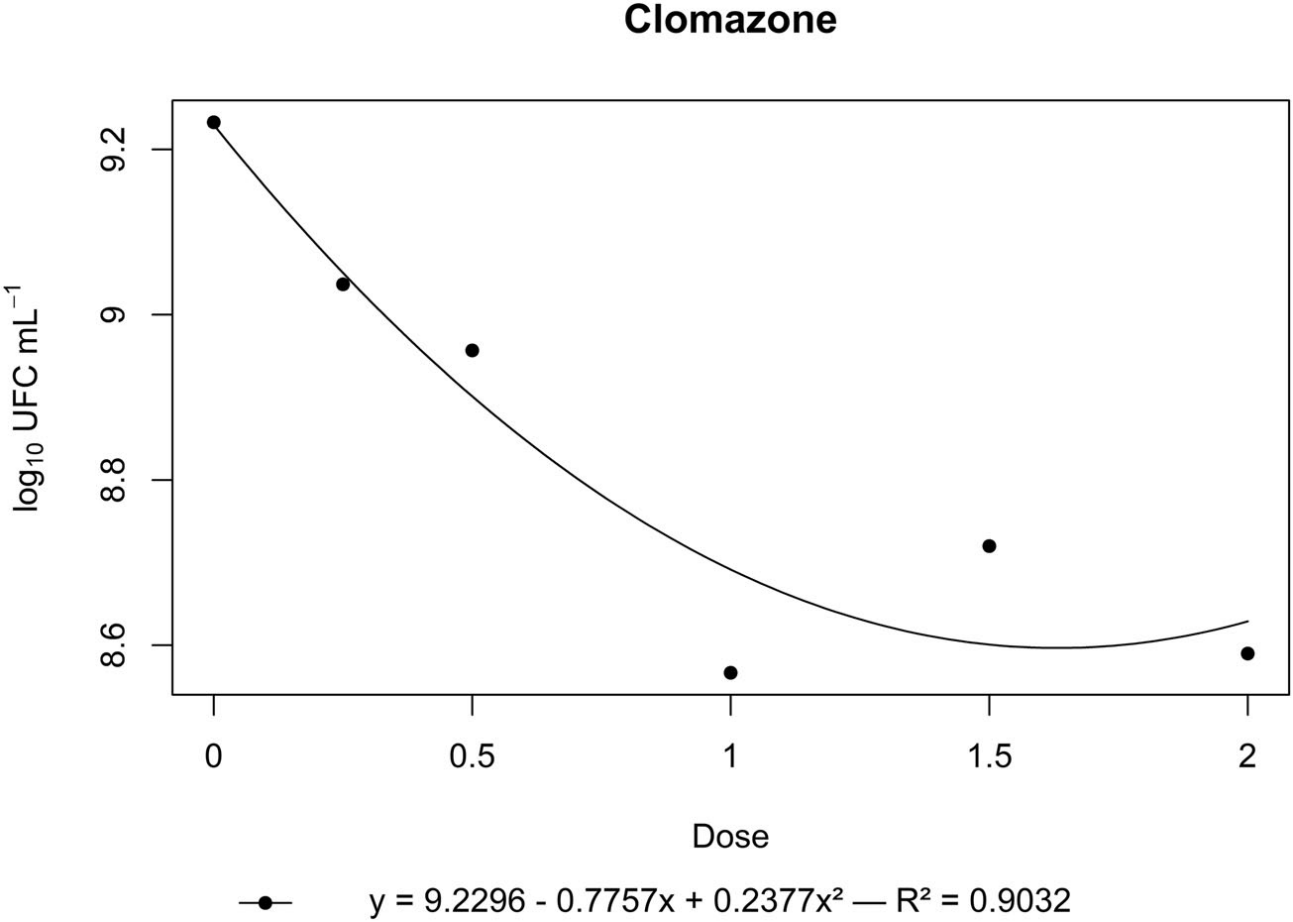
Log₁₀ of CFU mL^−1^ of *N. amazonense* grown in nutrient broth containing clomazone at 2×, 1.5×, 1×, 0.5× and 0.25× of the commercial dose of 720 g a.i. ha^−1^.

**Figure 2:**
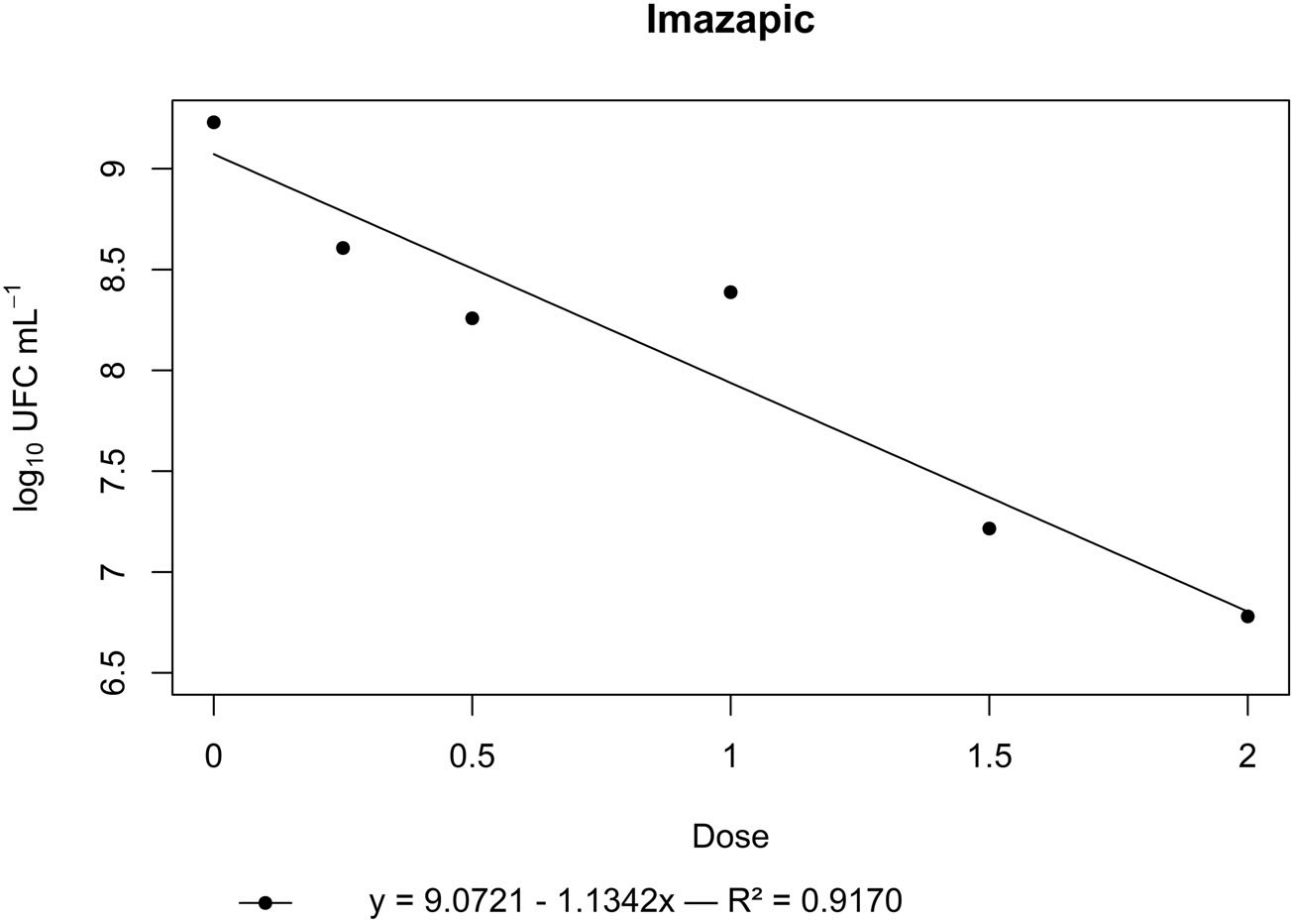
Log₁₀ of CFU mL^−1^ of *N. amazonense* grown in nutrient broth containing imazapic at 2×, 1.5×, 1×, 0.5× and 0.25× of the commercial dose of 200 g a.i. ha^−1^.

**Figure 3:**
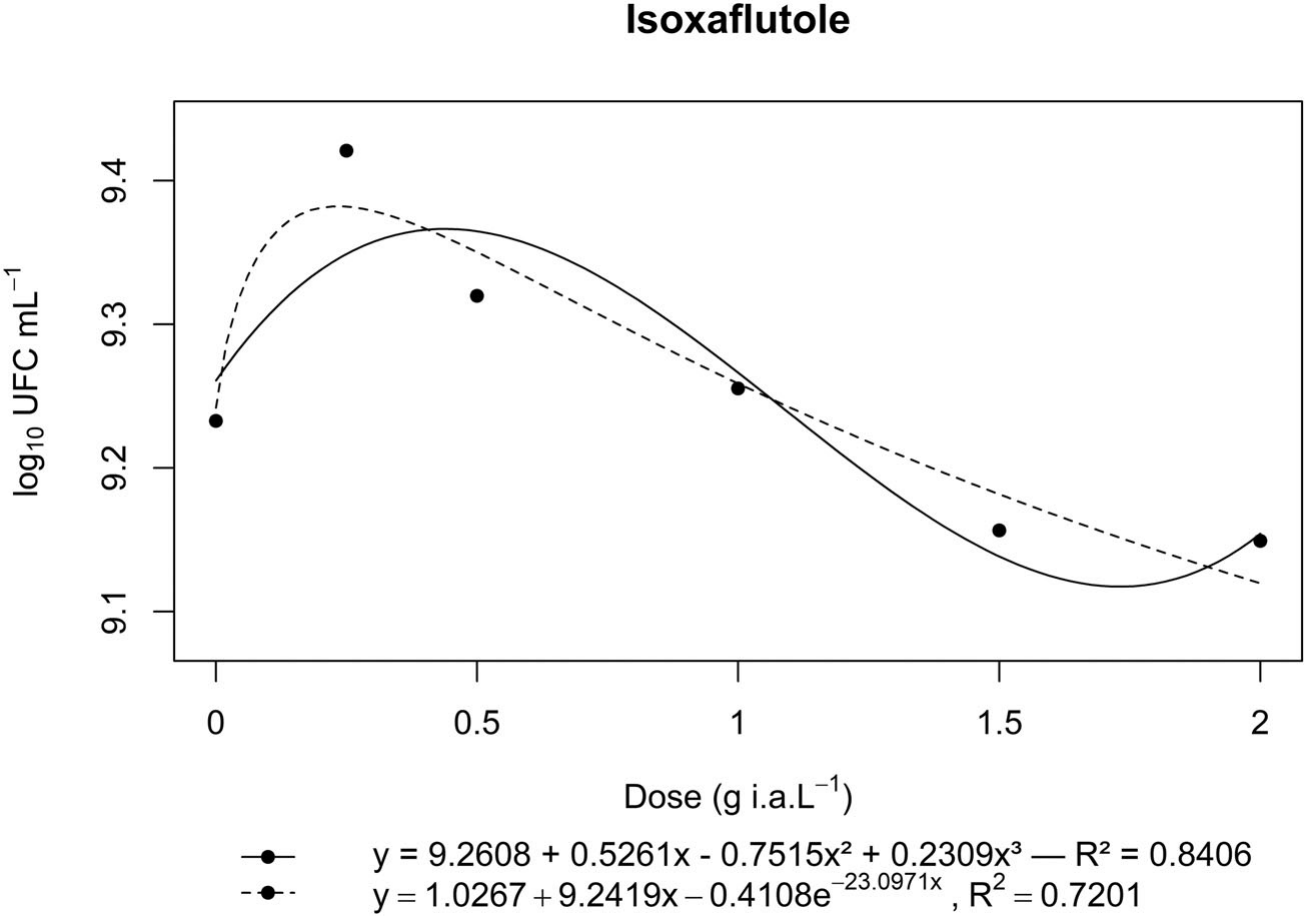
Log₁₀ of CFU mL^−1^ of *N. amazonense* grown in nutrient broth containing isoxaflutole at 2×, 1.5×, 1×, 0.5× and 0.25× of the commercial dose of 150 g a.i. ha^−1^.

**Figure 4:**
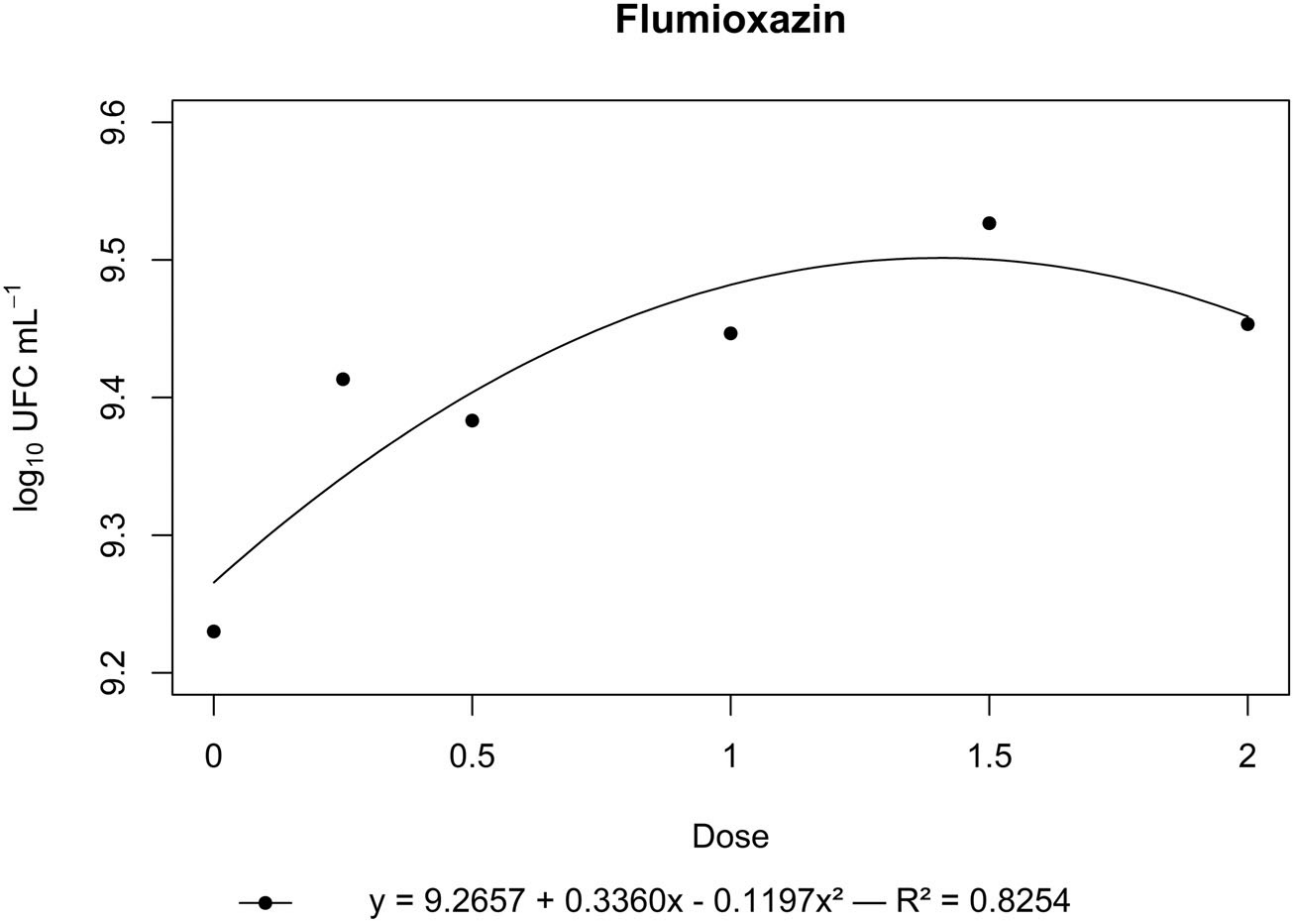
Log₁₀ of CFU mL^−1^ of *N. amazonense* grown in nutrient broth containing flumioxazin at 2×, 1.5×, 1×, 0.5× and 0.25× of the commercial dose of 150 g a.i. ha^−1^.

Clomazone was toxic to *N. amazonense*, causing a significant reduction in bacterial growth compared with that of the control, even at the lowest concentration tested (0.25× the recommended dose). Growth, measured in CFU mL^−1^, continuously decreased as the herbicide concentration increased to the 1.5× dose, after which it stabilized (Fig. 1).

**Figure 5:**
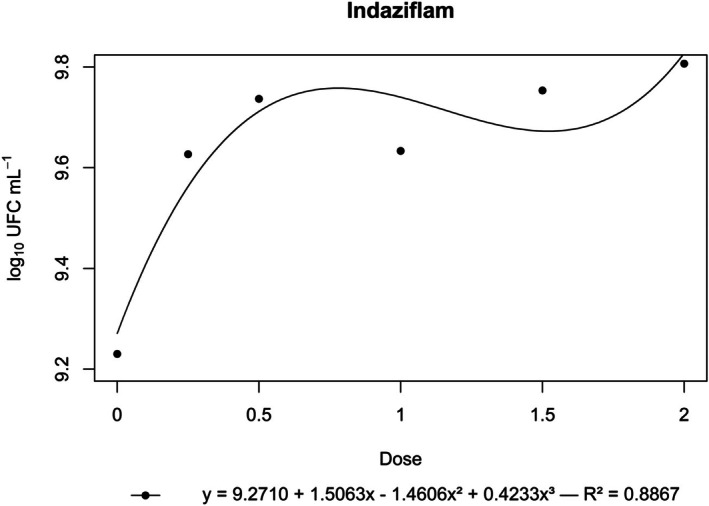
Log₁₀ of CFU mL^−1^ of *N. amazonense* grown in nutrient broth containing indaziflam at 2×, 1.5×, 1×, 0.5× and 0.25× of the commercial dose of 75 g a.i. ha^−1^.

**Figure 6:**
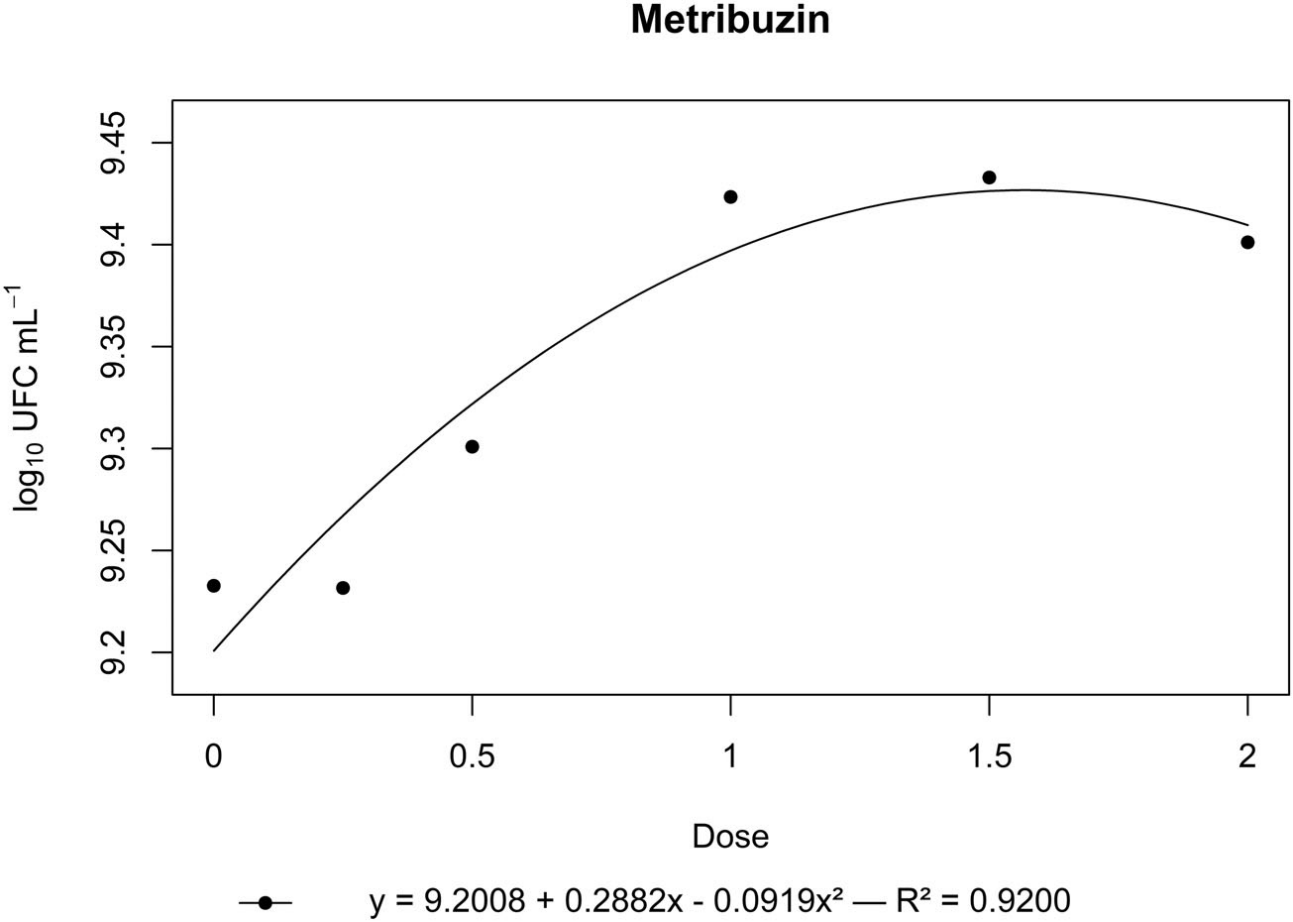
Log₁₀ of CFU mL^−1^ of *N. amazonense* grown in nutrient broth containing metribuzin at 2×, 1.5×, 1×, 0.5× and 0.25× of the commercial dose of 1680 g a.i. ha^−1^.

**Figure 7:**
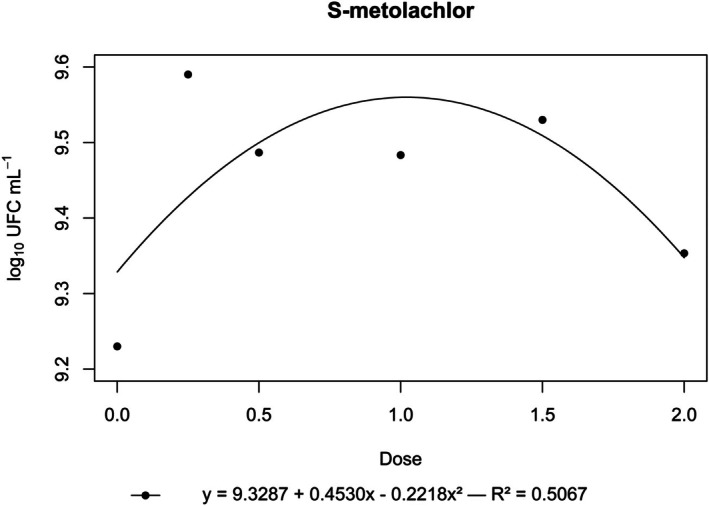
Log₁₀ of CFU mL^−1^ of *N. amazonense* grown in nutrient broth containing S‐metolachlor at 2×, 1.5×, 1×, 0.5× and 0.25× of the commercial dose of 1680 g a.i. ha^−1^.

**Figure 8:**
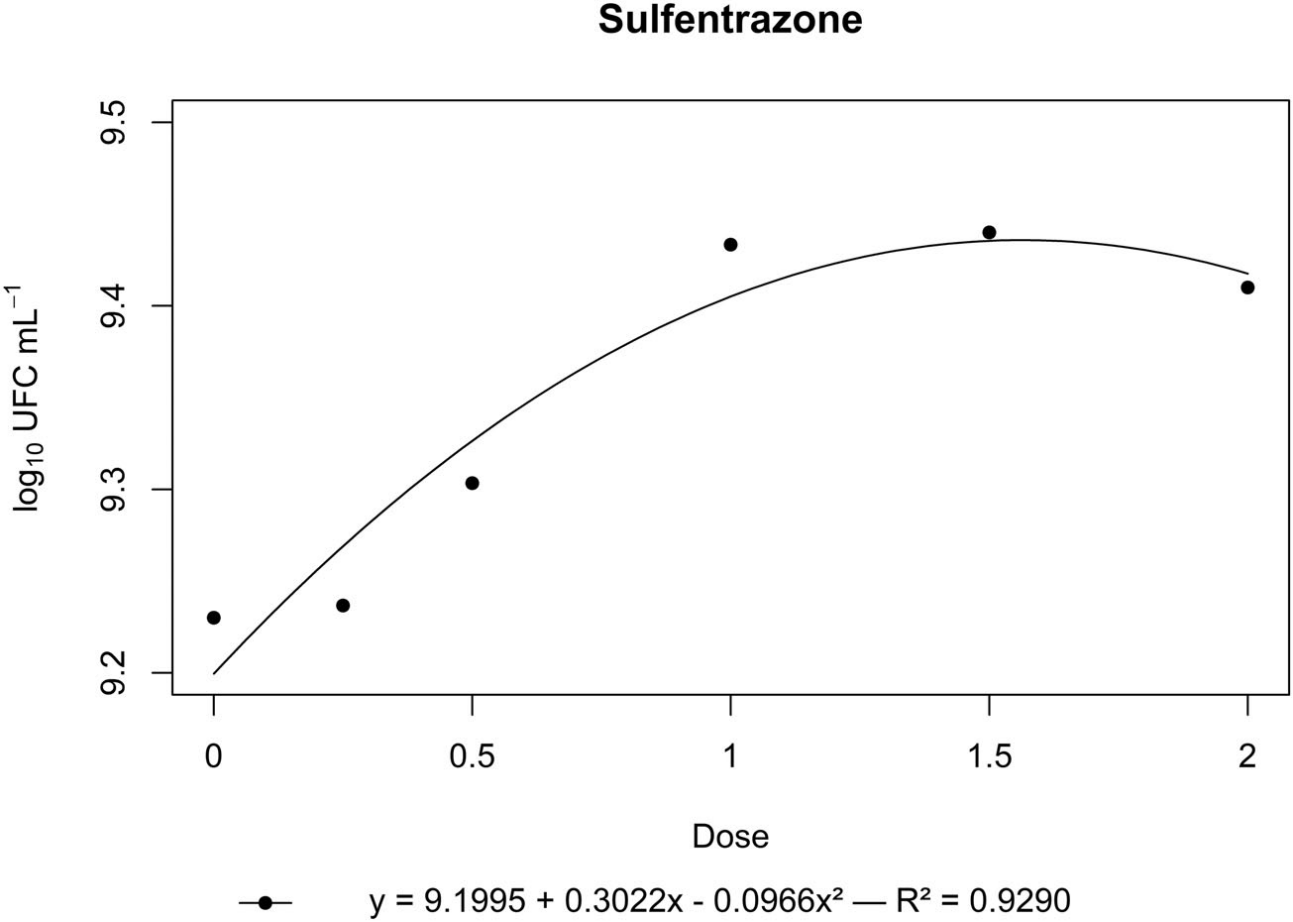
Log₁₀ of CFU mL^−1^ of *N. amazonense* grown in nutrient broth containing sulfentrazone at 2×, 1.5×, 1×, 0.5× and 0.25× of the commercial dose of 800 g a.i. ha^−1^.

Likewise, imazapic had an inhibitory effect. A significant negative linear relationship was observed between its concentration and bacterial growth, with the CFU mL^−1^ consistently declining as the dose increased (Fig. 2).

Isoxaflutole stimulated bacterial growth at lower concentrations (up to the 0.5× dose) but became toxic as the concentration increased. Compared with those of the control, significant reductions in CFU mL^−1^ were observed above the commercial dose (1×). The Brain–Cousens model was applied to assess the occurrence of hormesis; however, although the visual pattern suggested such an effect, the estimated parameters (*R* ^2^ = 0.72; *e*, *P* = 0.299; *f*, *P* = 0.306) did not reach statistical significance (Fig. 3).

By contrast, flumioxazin, indaziflam, metribuzin, *S*‐metolachlor and sulfentrazone stimulated bacterial growth. For these herbicides, growth surpassed that of the control, and a positive correlation was observed between dose and CFU mL^−1^ (Figs 4, 5, 6, 7, 8).

The peak bacterial growth for *S*‐metolachlor was observed at the commercial dose (1×). For flumioxazin, metribuzin and sulfentrazone, peak growth was reached at the 1.5× dose. The stimulatory effect of Indaziflam was maintained at the highest concentration tested (2× dose).

Tebuthiuron had no significant inhibitory or stimulatory effect. Across all of the tested concentrations, bacterial growth did not differ significantly from that of the control (Table 2).

**Table 2:** Log₁₀ of CFU mL^−1^ of *N. amazonense* grown in nutrient broth supplemented with increasing doses of tebuthiuron

Commercial dose	Log10 CFU mL^−1^
0	9.23 a
0.25	9.19 a
0.50	9.22 a
1	9.14 a
1.5	9.23 a
2	9.20 a
**Coefficient of variation** (%)	**1.35**

† CFU = colony forming units.

In the evaluation of herbicide toxicity in soil (Table 3), only isoxaflutole resulted in a significant reduction in the MPN of *N. amazonense* cells. All the other herbicides tested did not significantly affect bacterial survival under the conditions evaluated.

**Table 3:** Log₁₀ of the most likely number (MPN) of CFU g^−1^ of soil for *N. amazonense* following the application of different herbicides

Treatments	CFU g^−1^ of soil
Control (inoculum only)	7.15 a
*S*‐metolachlor	7.15 a
Metribuzin	7.15 a
Flumioxazin	7.15 a
Sulfentrazone	7.15 a
Indaziflam	7.15 a
Tebuthiuron	7.15 a
Imazapic	5.89 ab
Clomazone	5.54 ab
Isoxaflutole	4.17 b
Control (blank)	1.53 c
**Coefficient of variation** (%)	**14.53**

† CFU = colony forming units.

Although clomazone and imazapic led to numerically lower MPN values (log₁₀ CFU g^−1^ soil), this reduction was not statistically significant compared with that of the control (Tukey's HSD test, *P* > 0.05). Conversely, the herbicides *S*‐metolachlor, metribuzin, flumioxazin, sulfentrazone, indaziflam and tebuthiuron had no toxic effects on *N. amazonense* in soil, as their MPN values were statistically comparable to those of the control (inoculum only).

Table 4 shows the production of IAA by *N. amazonense* when cultured in media supplemented with each herbicide at its recommended commercial dose. Despite not affecting bacterial survival in either the culture medium or the soil, flumioxazin and tebuthiuron significantly inhibited the ability of bacteria to produce this phytohormone. By contrast, *S*‐metolachlor, sulfentrazone, clomazone and imazapic stimulated IAA production. Moreover, isoxaflutole, indaziflam and metribuzin did not significantly affect auxin production, with levels statistically equivalent to those in the control treatment.

**Table 4:** Indole‐3‐acetic acid production (mg L^−1^) by *N. amazonense* cultured in media supplemented with different herbicides

Treatments	IAA (mg L^−1^)
*S*‐metolachlor	22.22 a
Sulfentrazone	20.52 ab
Clomazone	17.92 ab
Imazapic	16.77 abc
Isoxaflutole	15.40 bcd
Indaziflam	14.84 bcd
Control (inoculum only)	14.56 bcd
Metribuzin	14.56 bcd
Flumioxazin	10.39 cd
Tebuthiuron	09.05 d
**Coefficient of variation** (%)	**14.72**

The BNF capacity of *N. amazonense* in media containing different herbicides is presented in Table 5. None of the herbicides tested negatively affected the BNF ability of the bacterial strain. Tebuthiuron, metribuzin, imazapic, sulfentrazone and isoxaflutole stimulated BNF by the bacterium. By contrast, flumioxazin, clomazone, indaziflam and *S*‐metolachlor were not significantly different from those in the control treatment.

**Table 5:** Biological nitrogen fixation by *N. amazonense* in media subjected to different herbicide treatments

Treatments	Nitrogen fixation (mg mL^−1^)
Tebuthiuron	0.44 a
Metribuzin	0.30 ab
Imazapic	0.26 bc
Sulfentrazone	0.24 bc
Isoxaflutole	0.18 bc
Flumioxazin	0.12 c
Clomazone	0.10 c
Control (inoculum only)	0.10 c
Indaziflam	0.10 c
*S*‐metolachlor	0.09 c
**Coefficient of variation** (%)	**30.48**

**Figure 9:**
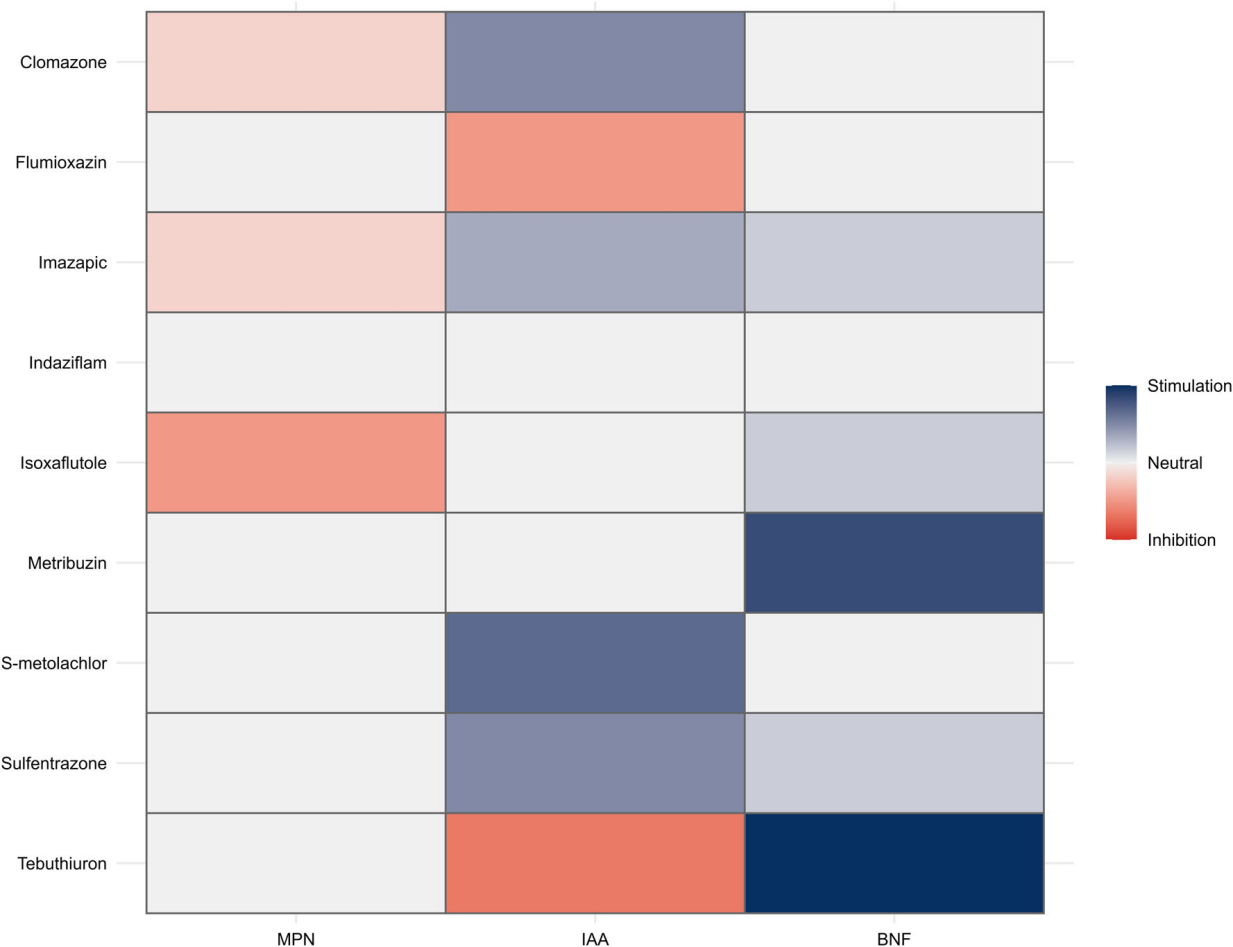
Heatmap of the effects of different herbicides on *N. amazonense*: soil survival (MPN), IAA production and BNF.

In summary, indaziflam, metribuzin, *S*‐metolachlor and sulfentrazone did not impair the survival or functionality of the bacterial strain. By contrast, these herbicides promoted significant cell growth and increased the IAA and/or BNF. Isoxaflutole, at sublethal concentrations, stimulated growth but reduced bacterial proliferation at the commercial dose, with no detectable impact on IAA or BNF production. Clomazone and imazapic suppressed bacterial growth while maintaining survival in soil, simultaneously promoting IAA and BNF production. Finally, flumioxazin and tebuthiuron increased cell growth and BNF activity but led to a reduction in IAA synthesis (Table 6; Fig. 9).

**Table 6:** Comprehensive overview of the effects of different herbicides on the survival and functionality of *N. amazonense*

	Assessments			
Herbicide	Survival (Culture medium)	Survival (Soil)	Indole‐3‐acetic acid (IAA) production	Biological nitrogen fixation (BNF) capacity
Clomazone	−	=	+	=
Imazapic	−	=	+	=
Tebuthiuron	=	=	−	+
Indaziflam	+	=	=	=
*S*‐metolachlor	+	=	+	=
Metribuzin	+	=	=	+
Isoxaflutole	+ −	−	=	=
Sulfentrazone	+	=	+	=
Flumioxazin	+	=	−	=

## 4 DISCUSSION

This study demonstrated that the evaluated herbicides had distinct effects on the survival and functionality of *N. amazonense*. The bacterial responses spanned a diverse spectrum, from metabolic stimulation and growth promotion to significant inhibition and acute toxicity. Notably, four herbicides—indaziflam (a cellulose synthesis inhibitor), metribuzin (a photosystem II inhibitor), *S*‐metolachlor (a long‐chain fatty acid inhibitor) and sulfentrazone (a PROTOX inhibitor)—exhibit favorable effects, simultaneously enhancing the growth of *N. amazonense* as well as its BNF and/or auxin biosynthesis activities. These findings highlight the potential compatibility of these herbicides with diazotrophic bacteria, suggesting their strategic use in integrated weed management systems for sugarcane cultivation.

Our results are well aligned with recent literature documenting the compatibility of specific herbicides with diazotrophic bacteria. Specifically, the lack of toxicity of indaziflam toward *N. amazonense* also was reported by Jonck *et al*.^15^ This is further supported by findings from Delgado *et al*.^16^ who documented no adverse effects on soil microbial respiration or biomass, and by Torres *et al*.^17^ who even demonstrated a stimulatory effect on the soil microbiota. Moreover, the observed tolerance is not limited to *N. amazonense*. Rabelo *et al*.^18^ recently demonstrated the high tolerance of *A. brasilense* to both sulfentrazone and indaziflam herbicides. In a comprehensive earlier study, Procopio *et al*.^13^ evaluated sulfentrazone, metribuzin, clomazone, isoxaflutole, imazapic and *S*‐metolachlor and concluded that these compounds did not impair the growth or nitrogenase activity of *A. brasilense*.

Conversely, a notable discrepancy concerning metribuzin has emerged in the literature. Several studies have documented its detrimental effects on diazotrophs of the *Azospirillum* genus, manifested as diminished microbial populations^19^, ^20^ and suppressed nitrogenase activity.^21^

We propose that these divergent outcomes stem from the distinct taxonomic identities of the microbial species under investigation. *Nitrospirillum amazonense* was recently reclassified from its former genus, *Azospirillum*, a critical distinction given that its toxicological profile in response to herbicides remains largely uncharacterized.^8^, ^22^ This fundamental interspecific variability underscores the ability to extrapolate toxicological data across different bacterial genera and reinforces the imperative for species‐specific assessments.

Isoxaflutole, a 4‐hydroxyphenylpyruvate dioxygenase inhibitor, demonstrated a distinct biphasic dose–response in *N. amazonense*. Sublethal doses (1/4 DC and 1/2 DC) elicited stimulation, whereas commercial doses were detrimental to bacterial growth and soil persistence. Paradoxically, this observed toxicity was decoupled from key metabolic functions, as neither AIA synthesis nor BNF was adversely affected. This finding resonates with the work of Petkova *et al*.,^23^ who reported that isoxaflutole‐degrading bacteria, including diazotrophs, could thrive even as the herbicide suppressed broader microbial communities. Collectively, our results challenge the assumption of a linear dose–response model, revealing a far more intricate and nonlinear interaction between isoxaflutole and its microbial targets. Although the present data did not provide statistically conclusive evidence of hormesis, the consistent biphasic pattern observed underscores the need for further investigation. Future studies with experimental designs specifically tailored to this purpose may help clarify the occurrence and underlying mechanisms of this effect in *N. amazonense*.^24^ Such an approach is warranted, as similar responses have frequently been reported for herbicides in microorganisms, particularly under nonenergy‐limiting conditions.^25^

Conversely, clomazone (a carotenoid synthesis inhibitor) and imazapic (an acetolactate synthase inhibitor) demonstrated remarkable functional resilience. Although both herbicides inhibited *N. amazonense* growth *in vitro*, this effect did not translate to reduced cell viability in the soil matrix. Strikingly, these compounds even increased the physiological output, increasing both IAA synthesis and BNF. These findings corroborate previous *in vitro* tolerance assays for these molecules^12^ but stand in contrast to studies reporting detrimental effects on the wider soil microbial.^26^, ^27^ This divergence underscores a potential specific tolerance mechanism in *N. amazonense* that is not present in other soil microorganisms.

In this study, flumioxazin and sulfentrazone, two PROTOX inhibitors with identical modes‐of‐action (MoAs), elicited markedly different toxicological responses in *N. amazonense*. Specifically, while sulfentrazone enhanced IAA production, flumioxazin suppressed IAA production. This apparent paradox highlights the complexity of microbial–herbicide interactions and underscores the role of formulation‐specific factors in modulating toxicity. Herbicides with the same active ingredient, when delivered in different commercial formulations, may have significantly variable impacts on microbial communities. Components such as adjuvants and surfactants, which are commonly added to formulations, can reduce surface tension, increase cellular permeability, and, in some cases, exert toxic effects that exceed those of the active ingredient itself.^28^ This dynamic was likewise noted by Santos *et al*.,^29^ who reported responses ranging from bacterial tolerance to total growth inhibition when nitrogen‐fixing bacteria were exposed to various commercial glyphosate formulations.

Therefore, it is imperative to underscore that the findings presented herein are intrinsically linked to the specific commercial formulations detailed in Table 1. The same active ingredients, when delivered in alternative formulations, could yield substantially different toxicological outcomes for the microbial strain.

This variability in the bacterial response can be attributed to the intricate network of biochemical and physiological processes governing BNF, phytohormone production and cellular viability. These processes are mediated by discrete metabolic pathways, each exhibiting differential sensitivities to xenobiotic compounds.^30^

The microbial response to xenobiotic exposure is mediated primarily by two key processes: metabolism and cometabolism.^31^ In this context, metabolism involves the catabolic breakdown of a pesticide through enzymatic oxidation–reduction pathways, enabling the microorganism to harness the molecule for energy and essential nutrients, thereby promoting cell proliferation.^32^ This metabolic utilization is the most plausible explanation for the observed growth stimulation in *N. amazonense* when exposed to indaziflam, metribuzin, *S*‐metolachlor and sulfentrazone, suggesting that these herbicides were effectively leveraged as substrates for bacterial growth. The successful metabolic utilization of these herbicides by *N. amazonense* cannot be attributed solely to their MoA or to general patterns of physicochemical properties, as evidenced by the contrasting responses to herbicides that share the same target or differ in their chemical characteristics. Rather, this ability arises from a specific interplay between the molecular traits of each herbicide—defined by its chemical structure and formulation—and the bacterium's inherent capacity to interact with the compound, recruiting particular catabolic pathways to exploit it as a nutrient source.^33^

However, before the herbicide degradation process can begin, microorganisms must first tolerate the oxidative stress induced by molecular toxicity.^34^ Through cometabolism, detoxification and degradation pathways lead to the biotransformation of toxic organic compounds that do not serve as energy sources or structural components for the cell.^35^

These adaptive responses and detoxification pathways are specific to microorganisms and the chemical structure of xenobiotic compounds and are also influenced by environmental conditions.^36^ Common microbial stress response mechanisms to pesticide exposure include the following: forming biofilms for cellular protection; developing cross‐resistance through efflux pumps that expel toxic molecules; upregulating and increasing the copy number of stress tolerance genes; acquiring multiresistant phenotypes via horizontal gene transfer; and activating antioxidant systems.^37^

These mechanisms enable microbial survival and growth, yet they can create an energetic imbalance that compromises other cellular functions. For example, in *N. amazonense*, although the bacterium efficiently metabolizes the herbicides flumioxazin and tebuthiuron, which support its growth, its production of IAA is compromised. A plausible explanation for this trade‐off is the redirection of metabolic resources toward herbicide degradation and detoxification, consequently impairing IAA synthesis.

Conversely, under stress, microbial cells can upregulate the expression of functional genes.^38^ For example, Ona *et al*.^39^ reported that a pause in the growth of *A. brasilense* is a prerequisite for the expression of genes involved in IAA production. This finding may explain the results for the herbicides imazapic and clomazone, which, despite reducing *N. amazonense* cell counts, stimulated increased IAA production.

Furthermore, an examination of the herbicides that were toxic to *N. amazonense* (imazapic, clomazone, tebuthiuron, isoxaflutole and flumioxazin) reveals a common pattern: with the exception of imazapic, all herbicides are predominantly nonionic lipophilic molecules.^40^ This suggests that their physicochemical properties are likely to have influenced the observed toxicity. Both lipophilicity and a neutral charge can affect cellular diffusion, as nonionized lipophilic substances interact more readily with cell membranes than their ionized counterparts do.^41^

Imazapic, in turn, is a weak acid and is only moderately lipophilic. Its toxicity to *N. amazonense* likely stems from its specific MoA: the inhibition of an essential amino acid synthesis pathway common to both plants and microorganisms.^42^

In summary, the results indicate that the strategic integration of herbicides and microbial inoculants represents a promising approach for sustainable sugarcane management. Within this context, indaziflam, metribuzin, *S*‐metolachlor and sulfentrazone emerged as herbicides compatible with *N. amazonense*, enhancing its growth and plant‐beneficial functions, and thus are prime candidates for safe co‐application. Conversely, herbicides that exhibited *in vitro* toxicity (clomazone, imazapic, isoxaflutole) or functional inhibition (flumioxazin, tebuthiuron) require caution. Although their use cannot be categorically excluded, careful management strategies are necessary. Translating these findings to field conditions will require further investigations to quantify potential agronomic trade‐offs and establish clear guidelines, whether through modified application timing, adjusted dosages, or avoidance of concomitant use with microbial inoculants when necessary. Ultimately, this study demonstrates that judicious herbicide selection is crucial, as it directly influences the success of microbial inoculations and the sustainability and effectiveness of integrated weed management strategies.

## CONFLICT OF INTEREST

The authors declare no competing interests.

## Data Availability

The data that support the findings of this study are available from the corresponding author upon reasonable request.
